# On the contribution of overt tactile expectations to visuo-tactile interactions within the peripersonal space

**DOI:** 10.1007/s00221-017-4965-9

**Published:** 2017-05-20

**Authors:** Manasa Kandula, Nathan Van der Stoep, Dennis Hofman, H. C. Dijkerman

**Affiliations:** Experimental Psychology, Helmholtz Institute, Utrecht University, Heidelberglaan 1, 3584 CS Utrecht, The Netherlands

**Keywords:** Peripersonal space, Overt tactile expectation, Vision, Touch, Multisensory integration

## Abstract

Since the discovery of neural regions in the monkey brain that respond preferentially to multisensory stimuli presented in proximal space, researchers have been studying this specialised spatial representation in humans. It has been demonstrated that approaching auditory or visual stimuli modulate tactile processing, while they are within the peripersonal space (PPS). The aim of the current study is to investigate the additional effects of tactile expectation on the PPS-related multisensory interactions. Based on the output of a computational simulation, we expected that as tactile expectation increases rapidly during the course of the motion of the visual stimulus, the outcome RT curves would mask the multisensory contribution of PPS. When the tactile expectation remains constant during the motion, the PPS-related spatially selective multisensory processes become apparent. The behavioural results on human experiments followed the pattern predicted by the simulation. That is, rapidly changing levels of tactile expectation, caused by dynamic visual stimuli, masks the outcome of the multisensory processes within peripersonal space. This indicates that both PPS-related multisensory interactions and tactile expectations play an important role in anticipating and responding to interactions with the body.

## Introduction

Since the initial discovery of multisensory neurons in the periarcuate cortex of monkeys (Rizzolatti et al. 1981), the multisensory representation of an ultra-near space surrounding the body has been the subject of extensive research in humans and their primate cousins. These multisensory neurons possess both visual and tactile spatial receptive fields (SRF). As such, these neurons respond to objects seen around a body part and to tactile stimulation of the same body part. Strikingly, the visual SRFs are anchored to the body part rather than a specific location in space, coding visual information in a body-part centred frame of reference (Fogassi et al. 1996; Graziano and Gross 1994; Graziano et al. 1997). Such multisensory neurons have also been observed in other cortical regions such as the putamen (Graziano and Gross 1993), ventral intraparietal area F4 (Colby et al. 1993; Duhamel et al. 1998), and in certain parts of the premotor cortex in monkeys (Batista et al. 1999).

Perhaps, one of the most salient properties of these multisensory neurons is the way that they represent the space proximal to a body part. The combined spatial extent of the visual^1^ SRFs of these bimodal neurons is often labelled the peripersonal space (PPS). These SRFs have been found to project several centimetres into the space abutting their respective body parts in all directions. Due to their close link to motor neurons in the premotor cortex, it has been postulated that the activity of these neurons sub serves the representation of a spatial location (that is signalled by the encroaching object) allowing a potential action directed towards that location (Fadiga et al. 2000; Fogassi et al. 1996). The stimulation of these neurons results in the production of stereotypical defensive responses to protect the body part in question (Cooke and Graziano 2004; Cooke et al. 2003).

These neurons are also highly sensitive to moving stimuli that are approaching a body part as compared to receding stimuli, responding more consistently and vigorously to the former (Graziano et al. 1997). That is, PPS neurons respond to stimuli looming towards a body part and to tactile stimuli delivered on that body part. When observers are asked to respond to tactile stimuli, which are delivered at different timepoints during a looming stimulus’s approach, the pattern of response times is found to be sigmoidal in nature (Canzoneri et al. 2012; Ferri et al. 2015; Noel et al. 2016; Taffou and Viaud-Delmon 2014; Teneggi et al. 2013). That is, the surmised multisensory summative properties of the PPS neurons would speed up responses (RTs) to tactile stimuli that are delivered when the looming stimulus is within the PPS. This sudden decrease in RTs within the PPS would, therefore, be best explained by a sigmoidal function, whose midpoint would be indicative of the boundary of the PPS (Canzoneri et al. 2012).

When a moving object approaches the body, in addition to triggering the multisensory PPS neurons that influence tactile processing, the impending contact with this approaching object also creates an expectation of an upcoming tactile event. Tactile expectation may affect both tactile processing (Van Ede et al. 2011) and motor preparation (Umbach et al. 2012), influencing the speed of motor output. Tactile expectation is lowest when a stimulus is at the beginning of its trajectory far from the body, and increases as time elapses and it approached the body. Therefore, multisensory interactions and their motor outcome to events within the PPS might be subject not only to the action of their dedicated neural regions, but also to factors such as tactile expectation. The aim of the current study is, therefore, to investigate the influence of tactile expectation and visuo-tactile interactions within the PPS.

The consistency with which a prior cue predicts an upcoming target stimulus (cue-reliability) is a factor that modulates tactile expectation and, subsequently, the response time to these tactile events (Haegens et al. 2011). That is, when the warning cue is highly predictive of an upcoming tactile stimulus, from the onset of the cue, the tactile expectation increases with time. When the cue is non-predictive, tactile expectation remains relatively low, and response times are less affected by the prior presentation of the cue.

**Fig. 1:**
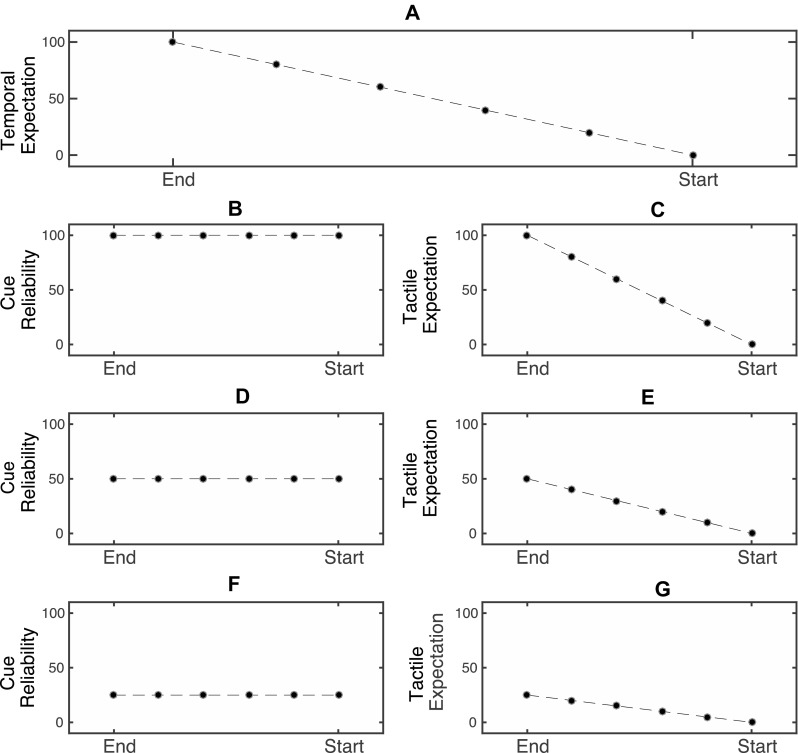
Expected interaction between temporal expectation levels at different timepoints during the trial and different levels of cue-reliability. The *x*-axis for all *plots* represents the timepoint within the trial. The *y*-axis represents the probability in percentage values. **a** Temporal expectation levels during a trial. These values are the same across all blocks. **b**, **d**, **f** Cue-reliability levels in different blocks. **c**, **e**, **g** Tactile expectation levels, resulting as the interaction between the temporal expectation level at different points in the trial and cue-reliability level in the block. When cue-reliability is high (**b**), the tactile expectation changes from a large value to a low value during the trial (**c**). When cue-reliability is low (**f**), the tactile expectation is low, and remains relatively uniform across the trial (**g**)

In our study, we used cue-reliability (the overall probability of receiving a tactile stimulus during a trial) as the factor to control the level of tactile expectation throughout the trial (explained below). That is, we expected that cue-reliability (Fig. 1b, d, f) and changes in temporal expectation (defined here as the expectation of a tactile stimulus based on the current location of the dynamic visual stimulus, Fig. 1a) would interact to produce the tactile expectation levels during the course of the trial. A low cue-reliability level can be used to maintain tactile expectation at a consistent level throughout the trial to largely isolate PPS-related multisensory effects. Thus, when the cue-reliability levels are low (Fig. 1f), tactile expectation should change less drastically during the course of the trial (Fig. 1g), remaining at a low level. When the cue-reliability is high (Fig. 1b), tactile expectation increases linearly (with a high slope) as the trial elapses (Fig. 1c).

Before we conducted a study in human participants, the interaction effects of PPS modulation and tactile expectation on response times to tactile stimuli were explored using a computational model. Following this, a tactile detection task involving approaching visual stimuli (Canzoneri et al. 2012) using three cue-reliability levels was run in separate blocks in human subjects. By analysing the response-time curves, we could determine the contribution of the effects of changing tactile expectation on PPS multisensory modulations. It was expected that when tactile expectation changes rapidly during stimulus motion, the resulting response-time patterns would be linear in nature. When tactile expectation is minimised and held at a constant level, the outcome response-time patterns should be sigmoidal in shape reflecting a PPS boundary.

## Methods

### Data modelling

To predict the changes in response-time patterns depending on the expectation of a tactile stimulus, a simple simulation model was constructed using the Matlab software (MATLAB and Statistics Toolbox Release 2015b, The MathWorks, Inc., Natick, Massachusetts, United States). The aim of this model was to gain insight into the changes of the response-time pattern of an imaginary observer to a tactile stimulus, while considering the current location and direction of motion of the visual distractor stimulus, and cue-reliability. It should be noted that the simulation model is intended only to approximate observable human behaviour and does not account for, or match the specific details of the underlying neural dynamics. We intended only to test if the shape of the outcome response-time patterns, and not if the absolute response times may be modulated by the aforementioned tactile expectation. We assume that the outcome response times are a result of a two-stage serial process consisting of a detection and response stage. We also assume that prior expectations directly and equally influence the amount of activation in each of these stages.

**Fig. 2:**
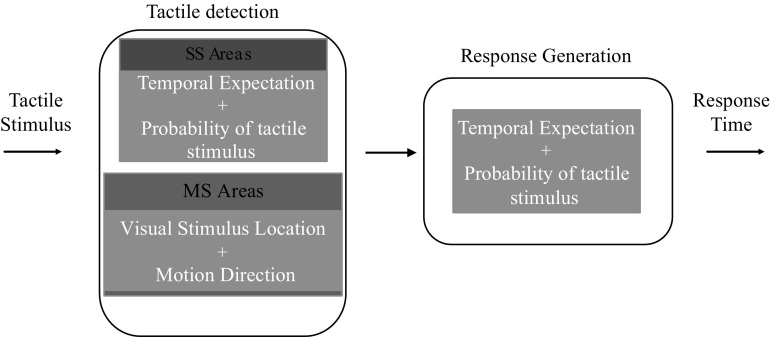
Illustration of the model’s stages. Once the tactile stimulus is delivered, the latency for the tactile stimulus to be detected is computed by the Tactile Detection stage (*left box*). Once the tactile stimulus is detected, the Response Generation stage (*right box*) of the model computes the latency with which a response to the tactile stimulus is generated. Both the stages are influenced by the expectation of the tactile event and cue-reliability

The data model consisted of two serial stages (see Fig. 2). The first stage computes the latency in detecting the tactile target. It considers the direction of the motion of the visual distractor and its distance from the observer. It also is affected by the changing temporal expectation (caused by the location of the visual looming stimulus) and cue-reliability levels (Fig. 2). The second stage computes the latency required for the observer to initiate a response. It is also influenced by the level of expectation generated by the cue-reliability and the location of the visual stimulus from the observer (temporal expectation) (Fig. 2).

### Detection stage

In this stage, once a tactile stimulus is delivered, the time taken for the tactile stimulus to be detected is dependent on the time taken for the underlying somatosensory module (SS) and PPS multisensory module (MS) together to reach a detection threshold. The activity of the somatosensory module is sensitive to temporal expectation, which is dependent on the distance travelled by the visual stimulus (distance travelled by the visual stimulus is indicative of the temporal specifics of trial) and not to its direction. The temporal expectation is further modulated by the cue-reliability level, or the overall probability of receiving a tactile stimulus. The MS module is insensitive to temporal expectation, but is influenced by the physical location of the visual stimulus and its movement direction. That is, if the visual stimulus is within the PPS zone and is moving towards the observer, the MS module contributes to the detection of the tactile stimulus.

The amount of activation (generated by the combined activity of the SS module and MS module) is dependent on the level of tactile expectation, which follows the following rules:

1. The amount of tactile expectation at a given timepoint is a factor of the overall probability of receiving a tactile stimulus (cue-reliability) for that condition and the location of the visual stimulus along its movement trajectory (temporal expectation) (Eq. 1).
2. The maximum amount of tactile expectation is determined by the cue-reliability level.
3. The minimum amount of tactile expectation calculated by dividing the maximum amount of tactile expectation by the number of timepoints during the motion of the visual stimulus, where a tactile stimulus may be delivered:

1 $${\text{TE}}_{{t{\text{current}}}} = A_{\text{threshold}} \times \frac{{P_{\text{tactile}} }}{100} \times \frac{{t_{\text{current}} }}{{t_{\text{total}} }}.$$

TE_*t*current_ is the tactile expectation when the tactile stimulus is delivered at timepoint *t*_current_, during the motion of the visual distractor. *A*_threshold_ is the activation threshold in units. *P*_tactile_ is the probability of receiving a tactile stimulus for that probability condition (cue-reliability). *t*_current_ is the current timepoint from the start of the motion of the visual stimulus. This is independent of the direction of the motion of the visual stimulus. *t*_total_ is the total duration of motion of the visual stimulus. For example, in the condition where there is a 100% probability of receiving a tactile stimulus, the amount of tactile expectation varies from 14.28 to 100, where 14.28 is the initial tactile expectation.

As the visual stimulus moves from the starting location to the end location (irrespective of direction of motion), the tactile expectation varies from the minimum value to the maximum value of tactile expectation range for that cue-reliability level. The amount of activation in the somatosensory module is proportional to the tactile expectation level. The model assumes that 100 units of activation are required to reach the threshold. The activation grows steadily at the rate of 0.5 units per millisecond. Based on this, given a zero-valued level of  tactile expectation, the time required to detect the presence of a tactile stimulus is 200 ms.

The multisensory module is sensitive to physical location of the visual stimulus and its direction of motion, responding to the tactile stimulus only when the visual stimulus is within a spatial limit with respect to the imaginary observer while approaching the observer. The combined activity of the somatosensory and multisensory modules determines the time taken for the tactile stimulus to reach a detection threshold. That is, when both the somatosensory and multisensory modules are triggered, the time taken for the system to reach the detection threshold is lower than when only the somatosensory module is triggered. When a visual stimulus is within the spatial extent of the multisensory module, the model receives a boost of 20 units of activation, thereby reducing the detection time by 40 ms. The boundary of this spatial extent is set to be at 40 cm from the imaginary observer and conditional upon the stimulus approaching the observer. Therefore, in the receding condition, the PPS-related multisensory gain is absent.

### Response stage

After the tactile stimulus is detected by the first stage of the model, the response stage of the model also requires reaching a response threshold to generate the final response. The response preparation in this stage is set to a starting level based on the level of expectation of a tactile event (the response generation level behaves the same way as the tactile expectation level of the somatosensory module of the tactile detection stage). Once the tactile stimulus is detected, this initial level of response preparation rises until it reaches the response threshold. Therefore, a high starting level of response preparation results in faster response times. Like the detection stage, the response stage requires 100 units of activation to reach the threshold. Activation grows steadily at the rate of 0.5 units per millisecond. The amount of initial activation is dependent on the amount of response preparation, which follows the same rules as that of the somatosensory module in the tactile detection stage.

The total time taken for the model to generate a response to the tactile stimulus is, therefore, the time elapsed between the start of the detection stage and the output of the response stage. In addition to the latency generated by the two stages of processing by the model (which indicates the time taken by the participant to initiate a motor response), an additional 150 ms is added to the final response time, to account for the time taken by the observer to mechanically complete the foot pedal press required to register the response. The reason for this additional step is to extract response times from the model that lie in a range comparable to that of real human participants.

The total duration of motion of the visual stimulus was set to 2300 ms, and there were seven timepoints (ranging from 0 to 2300 ms in steps of 328 ms) at which it was possible to receive a tactile stimulus. The visual stimulus was simulated to move between 0 and 90 cm from the subject in either an approaching, or receding fashion. Four ratio (cue-reliability) conditions were simulated. They were 100, 75, 50, and 25%. No noise was added to this system, so only one simulation per cue-reliability condition, timepoint, and motion direction were performed.

### Analysis

**Fig. 3:**
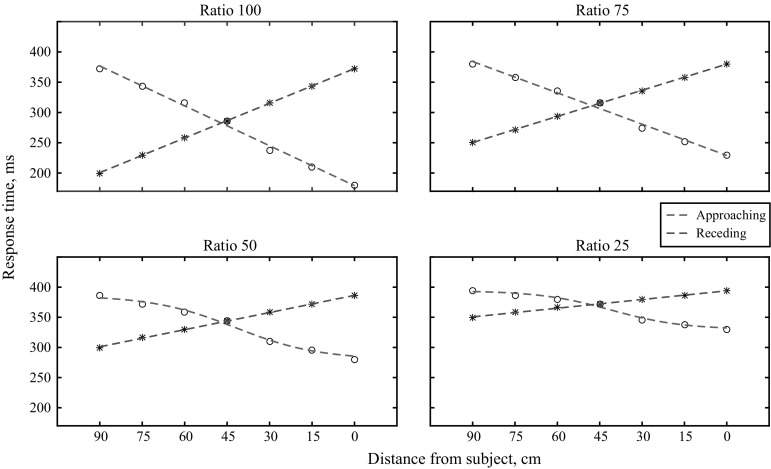
Data output of the model for different movement directions and ratio conditions. The best fitting curve is fitted onto the data points. The sigmoidal curve is the best fit only for the Approaching Ratio 25 and Approaching Ratio 50 conditions

The response times obtained from running the model simulations were fitted using a sigmoidal and a polynomial curve (Fig. 3). The sigmoidal fit was defined by the formula:

2 $${\mathbf{Y}} = {{y}}_{{{{min}}}} + \frac{{\left( {{{y}}_{{{{max}}}} - {{y}}_{{{{min}}}} } \right)}}{{1 + {\text{e}}^{{\left( {{{a}} - {\mathbf{L}}} \right) \times {{b}}}} }}.$$

**Y** is the vector of estimated response times from the curve-fitting procedure. *y*_*min*_ is the minimum response time obtained from the model simulation for that condition. *y*_*max*_ is the maximum response time obtained from the model simulation for that condition. **L** is the vector of locations of the visual stimulus at which the tactile stimulus is delivered. *a* and *b* are the factors estimated by the curve-fitting procedure. *a* is the location at which the response time reaches its mean value. It is treated as the location of the PPS boundary. *b* is the slope of the sigmoid curve.

A sigmoid curve was estimated for each cue-reliability condition (100, 75, 50, and 25%, and visual stimulus movement direction (Approaching, Receding).

The linear fit was defined by the following function:

3 $${\mathbf{Y}} = {{m}} \times {\mathbf{L}} + {{c}}$$

where **Y** is the vector of estimated response times from the curve-fitting procedure. *c* and *m* are the coefficients estimated by the curve-fitting function. *c* is the intercept of the curve. *m* is the slope of the linear curve. **L** is the vector of locations at which the tactile stimulus is to be delivered.

A total of eight linear curves were estimated for each ratio condition and visual stimulus movement direction (Fig. 3). Root mean square errors (RMSE, Eq. 4) were calculated for the obtained outcome curves for each of the ratio, direction conditions, and for the sigmoidal and linear fits. When comparing the estimated linear and sigmoidal fits, the fit with the lowest RMSE value more closely follows the obtained data:

4 $${\text{RMSE}} = \sqrt {\frac{1}{N}\mathop \sum \limits_{i = 1}^{N} \left( {{\hat{\text{Y}}\text{i}} - {\text{Yi}}} \right)^{2} } .$$

The RMSE value is calculated separately for each Ratio and Direction condition, and separately for both the sigmoidal and linear curves. Here, *N* equals the total number of data points used in each curve (*N* = 7 in our simulation, as RTs from seven different locations of the visual stimulus were used). ${\hat{\text{Y}}\text{i}}$ is the RT at a location i as estimated by the curve-fitting procedure; ${\text{Yi}}$ is the RT at location i as obtained from the simulation model.

## Results and discussion

A value of the RMSE for each curve indicates how closely the fitted curve follows the given data. Therefore, to learn which curve was a better fit for each ratio and direction condition, the RMSE values obtained for the sigmoidal and linear curve were compared (Table 1). As expected, the sigmoidal fit improved as the overall probability of tactile stimulation decreased. Only in the Approaching condition for Ratios 50 and 25 was the sigmoidal curve a better fit, as the RMSEs for the sigmoidal curves for these conditions were lower than their corresponding linear curves.

**Table 1:** RMSE values for the sigmoidal and linear curves fitted for each of the movement directions and ratio conditions simulated by the model

Ratio	25	50	75	100
Approaching				
Sigmoidal	3.52	4.86	6.80	8.04
Linear	5.60	5.80	5.89	5.80
Receding				
Sigmoidal	2.59	4.58	6.86	8.70
Linear	0.65	0.65	0.58	0.65

The curve with the lower error RMSE explains the data for that condition better

From this simulation, it appeared that reducing tactile expectation to 50% was sufficient to bring forward the sigmoidal pattern of RTs assumed to be driven by multisensory interactions within the PPS. It must be mentioned, however, that by changing the amount of multisensory PPS contribution in the model (say, for example, from 20 to 40 units), the number of tactile absent trials required to bring out the sigmoidal nature of the RTs also changes. Specifically, when the contribution of the PPS is higher, the number of the tactile absent trials required to reduce the effects of the tactile expectation becomes lower.

The aim of this simulation was to check the idea that increasing tactile expectation levels during the course of a trial could influence responses to multisensory stimulus in PPS. The actual ratio of tactile present trials necessary to cause the emergence of this effect in behaving humans can only be gleaned with experimental evidence. The following experiment was performed to test this.

## Experiment 1

### Methods

#### Participants

Fifteen subjects between the ages of 18 and 30 were tested (*M*_age_ = 22 years, SD = 3.3, 12 females, 13 right-handed, 2 left-handed). All had normal or corrected-to-normal vision. The experiment was performed according to the ethical guidelines laid down by the Declaration of Helsinki. The local ethical committee of the Faculty of Social and Behavioural Science, Utrecht University also approved the study. All the participants gave their informed consent prior to their participation in the study.

#### Stimuli and apparatus

**Fig. 4:**
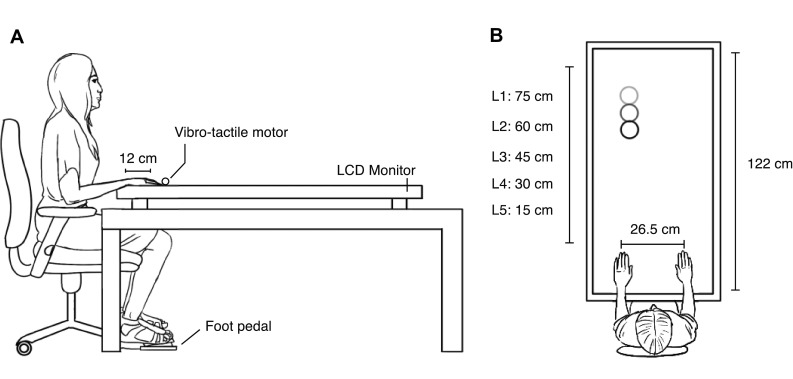
Schematic depiction of the experimental setup

The tactile stimulus was a 100 ms, 200 Hz vibration by a vibro-tactile motor (Precision Microdrives, Model: 308–100) that was attached to the distal phalanx on the back of both their left and right index fingers. The visual stimulus was a red dot with a diameter of 5 cm. The background of the screen was black in colour. The visual stimuli were displayed on large screen monitor (Philips BDT5530EM/06) lying flat on a table in front of them (see Fig. 4). The screen was 122 × 68 cm in size.

#### Procedure

The experiment took place in a darkened room in the Experimental Psychology lab at Utrecht University. Each subject was seated in front of the screen where the visual stimuli were displayed. The head rested on a chin rest at a height of 18 cm from the screen to maintain a consistent viewing height across all participants. They were instructed to place their hands on the edge of the monitor that was closest to them, such that each of their index fingers were 12 cm along the length the screen (see Fig. 4). Prior to the start of a block, an indicator dot was presented on each side of the screen at this distance, and participants were instructed to cover this dot with their index fingers. This ensured all participants consistently placed their fingers at the correct distance. Responses were given by depressing the foot pedal with the right foot when they detected a tactile target on the left or the right hand. In this position, they performed three blocks, each pertaining to a specific cue-reliability condition. The numbers of catch trials (no tactile target) were varied in each block. The order of the conditions was counterbalanced across subjects. In all other respects, the stimuli and procedure remained the same across all blocks.

Each trial began with the presentation of the visual stimulus on a black background appearing either at the far end of the screen (90 cm away from the tip of the index finger) or at the near end of the screen near the index finger (0 cm from the tip of the index finger), on the left or the right side of the screen (13.25 cm from the body midline). The visual stimulus remained there for a variable period between 400 and 800 ms after which it started moving. If the dot was presented at the far end of the screen, it moved towards the subject’s left or right finger in a straight line, and vice versa. Visual stimuli did not cross the midline of the screen during movement and only moved towards the finger at the same side as the visual stimulus.

From the start of the trial, there were seven locations in depth at which a tactile target could be delivered. In two conditions, the tactile stimulus was delivered when the stimulus was stationary at the far or the near end of the screen (*L*_far_ and *L*_near_, respectively). The purpose of these two locations was to encourage subjects to monitor the tactile modality throughout the duration of the trial. The visual locations of interest were L1–L5, and were delivered during the motion of the visual stimulus. On any target present trial, only one tactile stimulus could be delivered. Each location at which a tactile stimulus could be delivered was separated by 15 cm on the screen (distances ranged from 75 to 15 cm from the tip of the index finger, in steps of 15 cm). The total duration of visual motion was always 2300 ms.

Within each cue-reliability block (henceforth called the Ratio block for brevity), there were two direction conditions (approaching, receding), seven locations of the visual stimulus in depth at which the tactile stimuli may be delivered, two laterality conditions (left, right), and two target conditions (tactile-target present, tactile-target absent). The conditions were presented in a random order.

Based on the ratio, the total number of trials in the block varied. In each of the ratio blocks, each condition (two directions, seven visual locations, and two laterality conditions) was repeated approximately three times (the total number of trials per condition were rounded up to ensure a perfect ratio distribution). The experiment yielded a total of 168 conditions (3 Ratios, 2 directions, 7 locations, 2 laterality, and 2 presence of tactile stimulus).

The 25% ratio condition contained 84 target present and 252 catch trials, the 50% ratio condition contained 112 target present trials and 112 catch trials, and the 75% ratio condition contained 84 target present trials, and 28 catch trials. After every 112 trials, the participants received a 2-min break. As a result, there were two breaks in 25% ratio condition (3 sub-blocks), one break in the 50% ratio condition (2 sub-blocks), and no break in the 75% ratio condition (1 sub-block). Before the start of a ratio block, a practice block of 15 trials was presented to acclimatise the subject to the ratio contingency. No explicit instructions regarding the ratio were given. The subjects were headphones with continuous white noise playing to mask the sound of the vibrating motors. The entire experiment took approximately 45 min.

## Results and discussion

### Data analysis

#### Accuracy

All subjects had less than 10% false alarms during catch trials (*M* = 6.3%, SD = 0.82%). The number of missed presses in the *L*_near_ and *L*_far_ conditions was also analysed. Subjects missed less than 15% of tactile targets on target present trials (*L*_near_: *M* = 3.6%, SD = 2.71%, *L*_far_ = *M* = 5.16%, SD = 3.4%). All subjects missed less than 10% of the total amount of trials (*M* = 3%, SD = 1.647%). All subjects were therefore included for further analysis.

#### Response times

**Fig. 5:**
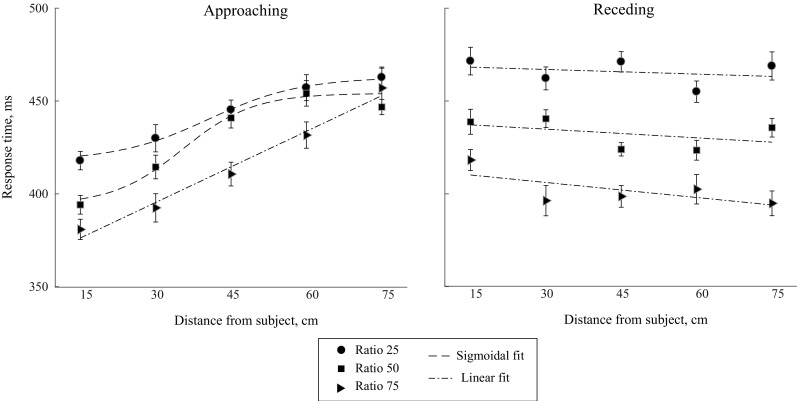
Average response times to the tactile targets at different locations of the visual stimulus during motion, along with their best fitting functions. For the approaching condition at ratio 25 and 50, the sigmoidal fit is the best representation of the data. The linear fit is the best representation for the rest of the conditions. The *error bars* for each condition were created by calculating the standard error of the mean after correcting for between-subject differences in the scores

The average response times per subject for each ratio, direction, laterality, and location condition (L1–L5) were calculated (see Fig. 5). The response times of all subjects fell within three standard deviations from the mean.

The laterality condition was collapsed onto a single factor for each subject. The average response times per subject for each ratio, direction and location condition were fed into a 3 × 2 × 5 Repeated Measures ANOVA. The factors were Ratio (75, 50, 25), Direction (approaching, receding), and visual location during tactile stimulation (L1–L5).

There was a main effect of Ratio [*F* (1.86, 26.06) = 15.488; *p* < 0.001, *n*_*p*_^2^ = 0.525]. Pairwise comparisons between the three ratio conditions revealed a significant difference between all conditions. RTs in the 50% condition (*M* = 436.25, SD = 55.58) were significantly faster than in the 25% condition [(*M* = 456.35, SD = 59.33) (mean difference = −20.10, *t* (14) = −2.779, *p* = 0.029). RTs in the 75% condition (*M* = 413.52, SD = 50.006) were significantly faster than in the 50% condition (mean difference = −22.7, *t* (14) = −2.787, *p* = 0.028). In addition, 75% was faster than the 25% condition (mean difference = −42.7, *t* (14) = −5.566, *p* < 0.001). The results indicated that the response times for ratio 75 were the shortest and increased as number of catch trials in the block increased (RTs: Ratio 75 < Ratio 50 < Ratio 25). We expect that, as the expectation to receive a tactile stimulus is higher in the higher ratio conditions, the preparation to respond would also have been higher in these conditions resulting in RTs in the higher ratio conditions being faster.

The effect of location was significant [*F* (2.38, 33.38) = 10.99; *p* < 0.001, *n*_*p*_^2^ = 0.440]. RTs increased with the distance from the subject (Table 2). Post hoc comparisons, corrected for multiple comparisons, showed some significant differences. RT_15_ < RT_60_ (mean difference = −17.11, *t* (14) = −3.992, *p* = 0.002), RT_15_ < RT_75_ (mean difference = −24.1, *t* (14) = −5.624, *p* < 0.001), and RT_30_ < RT_75_ (mean difference = −21.68, *t* (14) = −5.06, *p* < 0.001).

**Table 2:** Mean and standard deviation values for the response times to the tactile stimulus at different locations of the visual stimulus

Distance (cm)	Mean	Std dev
15	420.05	53.87
30	422.47	54.55
45	431.59	58.63
60	437.16	52.05
75	444.16	52.71

There was a significant interaction between ratio and direction [*F* (1.53, 21.42) = 15.47; *p* < 0.001, *n*_*p*_^2^ = 0.525], and between direction and location (*F* (2.42, 33.95) = 17.37; *p* < 0.001, *n*_*p*_^2^ = 0.553). As the goal of the study was to understand the nature of the tactile response-time patterns with respect to visual stimulus location for every ratio condition, and motion direction, we did not further analyse the interaction effects. Instead, we deferred their interpretation to the curve-fitting procedure.

#### Curve fitting

For this procedure, each participant’s response times obtained during the motion of the visual stimulus (L1–L5) were submitted into a curve-fitting procedure. In this process, the best fitting sigmoidal and linear curves were fit to the mean response times of each subject, for every ratio and direction condition. The equation used for the sigmoidal and linear curves is found in Eqs. (2) and (3), respectively.

To compare the fits of the sigmoidal and linear curves for each condition, *t* tests on the RMSE values obtained for each pair of sigmoidal and linear curves were performed (Eq. 4). Testing the RMSE values for the sigmoidal and linear curve pairs for the individual conditions allowed us to index which of the two models was a better fit for each condition. The curve that results in the significantly lower RMSE value is the better fit for that condition. To test this, we first compared for each condition if the RMSE values for the sigmoidal and linear fits differed significantly. If that was the case, we proceeded to check which of the two fits had the lower RMSE value and, therefore, was the better fit.

Significant differences in RMSE were found between the linear and sigmoid fits only in the approaching condition for the 50% ratio (mean difference = −2.139, *t* (14) = −2.78, *p* = 0.015) and ratio 25% (mean difference = −1.574, *t* (14) = −2.3, *p* = 0.03). The sigmoid-RMSEs were significantly lower for these two ratio conditions (approaching ratio 50 RMSE: *M*_Sigmoid_ = 19.4, SD = 14.2, *M*_Linear_ = 21.5, SD = 13.7; approaching ratio 25 RMSE: *M*_Sigmoid_ = 22.04, SD = 12.74, *M*_Linear_ = 23.6, SD = 12.46). There were no differences between the RMSE values for the linear and the sigmoid fit in the 75% condition for both approaching (mean difference = 2.9, *t* (14) = 0.54, *p* = 0.59) and receding directions (mean difference = 0.40, *t* (14) = 0.35, *p* = 0.73). In addition, for the 25 and 50% ratio receding curves, there were no differences between the RMSEs for the sigmoidal and linear fits (receding ratio 25: mean difference = 0.56, *t* (14) = 0.27, *p* = 0.79; receding ratio 50: mean difference = 5.6, *t* (14) = 1.21, *p* = 0.24).

To test if the midpoint of the sigmoid (reflecting the boundary of the PPS representation) differed between ratio conditions, we tested the boundaries for those conditions in which the sigmoidal curve was a better fit. There were no significant differences between the midpoints of both curves indicating that the boundary of PPS remained the same despite the probability of overt tactile expectation (ratio 25: *M* = 47.15 cm, SD = 8.6, ratio 50: *M* = 41.20, SD = 6.6, *p* = 0.072).

The results of the current experiment revealed that only in the approaching condition for ratios 50 and 25% was the sigmoidal curve a better fit than the linear curve. For the approaching 75 ratio condition, the fits of the sigmoidal and linear curves did not differ and the linear model is sufficient to explain the data.

For the receding condition, for all ratios, there were no significant differences between the fits of the linear and sigmoidal curves, therefore, making the fit of the linear model the default explanatory function for the data.

## General discussion

The aim of this study was to investigate the role of tactile expectation on PPS-related visuo-tactile processes. To do so, the shape of the response-time curves to tactile targets as a visual stimulus approached or receded from the site of tactile stimulation was compared between three different cue-reliability conditions. The reason for this manipulation is that tactile expectation would be low at the start of the stimulus motion, and increase linearly as the stimulus reaches the end of its trajectory. Therefore, to manipulate tactile expectation, we varied cue-reliability, or the probability to receive a tactile stimulus during a trial. In conditions where we wanted tactile expectation to play a role, we set cue-reliability at a high level (75%). When we wanted to limit the influence of tactile expectation, a low level of cue-reliability was used (50, or 25%). It was expected that non-predictive and counter-predictive cues could reduce the overall level of tactile expectation and keep them at a more uniform level during the course of the trial.

First, we used simulated data from a model that accounted for the interplay between PPS modulation on multisensory interactions and tactile expectation. It was observed that when the visual stimulus approached the subject, the resulting response-time pattern was linear (with a high slope) when tactile expectation was high. These results indicate that the linear effect of tactile expectation on RTs masks any multisensory effects of the PPS space. When tactile expectation was reduced, and maintained at a constant level throughout the motion of the visual stimulus (using low cue-reliability), the visuo-tactile interactions specific to the PPS remained. In other words, during low cue-reliability conditions (50 and 25% chance of a tactile target), the pattern of the response times was better explained by a sigmoidal function than a linear function. In the receding conditions, at all cue-reliability levels, the linear function was a better fit.

To test the predictions of the model, an experiment was conducted with human participants, in which they had to respond to a tactile stimulus delivered to their left or right finger-tip, while a task-irrelevant dynamic visual stimulus either approached or receded from them. The tactile stimulus was delivered at one of several timepoints during the motion trajectory of the visual stimulus or not at all during catch trials. To induce different ranges of tactile expectancies across different blocks, the visual cue-reliability was varied across blocks (ratio: 75, 50, 25%). In accordance with the simulation, the RT pattern in the behavioural data was better explained by a sigmoidal function, but only in the approaching condition, for the 50 and 25% ratios. In the receding conditions, our behavioural data did not show any PPS-related multisensory modulations, which is in line with the observation that PPS is mainly responsive to stimuli approaching the body (e.g., Graziano et al. 1997). Overall, the behavioural findings were in line with our model suggesting that tactile expectation can strongly influence the pattern of response times to multisensory stimuli within the PPS.

Based on the seminal work of Graziano and colleagues (Graziano and Cooke 2006), one of the functional roles of the PPS space network is to support object avoidance while navigating the environment. Therefore, PPS may be seen as a part of a larger defensive network, whose outer spatial boundary is the flight zone. While the encroachment of the flight zone by a threatening object may result in fleeing behaviour, the intrusion into PPS should result in a defensive response specific to the endangered body part. Seen in this light, in most cases, the PPS action might be limited to very sudden onset events occurring in proximal space, which require quick and automatic motor responses. Had the intrusion been detected sooner, the agent might either choose to flee the situation in the presence of real danger, or overtly modify his motion path to avoid contact.

One could wonder what the role of PPS is when the effects of tactile expectation are so evident in response-time patterns during predictable visual motion towards the body. However, when the abrupt appearance of an object triggers an avoidance action (typical to PPS), it is reasonable to assume that tactile expectation is absent. Therefore, tactile expectation would not influence PPS-related multisensory interactions and motor output in such cases. In contrast, there are also many situations where one might need to engage with looming objects that will eventually encroach the PPS (catching a ball, or responding to tactile stimuli in this experiment, for example). In these situations, the stereotypical protective actions of PPS need to be suppressed so that the goal-specific motor output may be initiated. It is in these situations that tactile expectation is likely to affect PPS space-related sensory-motor processes.

Previous studies have shown that the subjective hazard rate (the conditional probability that an event will occur given that it has not yet occurred after accounting for the uncertainty in time perception) of the upcoming stimulus is a factor that greatly influences the anticipation of the stimulus (Bueti et al. 2010; Janssen and Shadlen 2005).

The objective hazard rate of the stimuli in our study is given by Eq. (5):

5 $$h\left( L \right) = \frac{{P_{\text{tactile}} }}{{L_{\text{Total}} {-} L + 1}}.$$

Here, *h*(*L*) is the hazard rate at location *L*, and $L_{\text{Total}}$ is the total number of locations where the tactile stimulus may be delivered. *P*_tactile_ is the overall probability of receiving a tactile stimulus in the trial. The objective hazard rates in our study in the 100% condition (including the static locations), therefore, are 1/7, 1/6…. 1/1, which are non-linear in relationship. However, the behavioural response times where tactile expectation plays a role (for example, in the ratio 75% condition), seem to be linearly changing in our study. As our subjects’ temporal expectation is additionally informed by the location of the looming stimulus, which is less permeable to perceptual biases, a mathematical description of the subjective hazard rates, which are expected to directly influence tactile expectation, is more difficult to arrive at in our study. In the absence of this information, a linear approximation seems to fit the behavioural outcomes.

It must also be noted that given our design, it is difficult to disentangle the different sources of influence that tactile expectation provides. For instance, the expectation of the vibro-tactile stimulus may have directly influenced tactile processing (as seen in the case of Haegens et al. 2011) and/or motor preparation. Their action was modelled in the simulation as being additive in nature.

Our results demonstrate that the response to multisensory stimuli in PPS is influenced by the changing tactile expectation levels that accompany a looming stimulus. By reducing the cue-reliability levels, we could maintain the level of tactile expectation at a relatively low, and constant level. In this case, the multisensory interactions within the PPS became visible in the outcome response-time patterns of subjects. Therefore, future studies interested in delineating the boundary of PPS using multisensory interactions might benefit from using a low cue-reliability level to filter out the unwanted effects of tactile expectation on multisensory response times. This method has the drawback of lengthening the experiment due to the addition of a large number of catch trials that are uninformative to the study’s aims. However, such a curve-fitting procedure does offer the advantage of demonstrating the boundary between two spatial zones. However, when testing additional properties of the PPS boundary for body parts that have already shown to possess such a boundary in previous studies, a more economical approach would be to use the paradigm used by Noel and colleagues (Noel et al. 2015; Serino et al. 2015).

In these studies, the basic experimental task is like the one used in this study. However, instead of looking for evidence of a boundary using mathematical functions during the analysis, the authors look for evidence of multisensory response enhancements at different locations from the body using a tactile-only condition as a baseline. The drawback of this method is, however, that it cannot demonstrate that the change between the physical locations (where there is multisensory enhancement versus where there is none) is non-continuous in nature. That is, even if there is a linearly changing relationship between visual stimulus location and the response time, this method will likely indicate the presence of the boundary. Therefore, we believe that it might be best to reserve this method for body parts where a more vigorous method such as the one used in this study and previous studies has previously demonstrated a multisensory boundary for PPS (Canzoneri et al. 2012; Ferri et al. 2015; Taffou and Viaud-Delmon 2014).

In conclusion, tactile expectation and multisensory interactions in PPS may both play an important role in anticipating and responding to objects that may approach the body. As demonstrated here, their relative contributions likely depend on the nature and the expectation of the interaction.

## Footnotes

1 Although the visual and tactile response properties of PPS have been more extensively studied, there have also been reports of body-part centred responsiveness to auditory stimuli in these neurons (Graziano et al. 1999).
